# Multi-Dimensional Resources Management with GNN for Adaptive Routing Optimization

**DOI:** 10.3390/s26051530

**Published:** 2026-02-28

**Authors:** Judi Zhao, Haibo Pu, Jun Li, Ailin Chen, Jian Song

**Affiliations:** 1 College of Information Engineering, Sichuan Agricultural University, Ya’an 625014, China; zhaojudi@stu.sicau.edu.cn (J.Z.); lijun@sicau.edu.cn (J.L.); 2 Faculty of Information Engineering and Automation, Kunming University of Science and Technology, Kunming 650500, China; 202312201133@stu.kust.edu.cn (A.C.); songjian@kust.edu.cn (J.S.)

**Keywords:** routingoptimization, deep reinforcement learning, graph neural network, dynamic resource adaptation

## Abstract

The primary challenges in routing optimization include adapting to dynamic environments with frequent node and link changes. Handling the computational complexity of large-scale networks and balancing communication resources in multi-objective optimization are also key difficulties. Traditional methods focus on optimizing a single dimension, which limits their effectiveness in dynamic network environments. They often fail to capture the full network state, making it difficult to adapt to changes in topology or traffic. This lack of a comprehensive view leads to poor resource balance and suboptimal performance as network conditions change. To address these challenges, we propose an Adaptive Routing algorithm with joint optimization of Multi-dimensional network Resources (AR-MRs) based on graph neural networks (GNNs). This algorithm optimizes multiple network resources simultaneously and effectively tackles the issues of incomplete resource consideration and insufficient balance prevalent in existing routing methods. As a result, overall network performance and reliability are enhanced. Additionally, we innovatively design a resource-adaptive module based on GNN. By leveraging GNN, complex relationships between network links and nodes are captured. This module thoroughly analyzes network states and dynamically adjusts resource allocation. Balanced optimization across multiple resource dimensions is thereby ensured. The effectiveness of the algorithm was validated through deployment in a simulation environment. Simulation results indicate that, compared to existing solutions, this approach significantly reduces end-to-end communication delay, decreases bit error rate, and enhances packet transmission efficiency.

## 1. Introduction

The rapid advancement of emerging information technologies, including virtual reality, 4K+ video, online conferencing, and cloud services [1,2], has led to exponential growth in application demands. This surge presents significant challenges to the development of internet communications. Ensuring timely and reliable data transmission in communication networks necessitates efficient routing optimization. Traditional routing algorithms, including Dijkstra’s algorithm [3], the Bellman-Ford algorithm [4], and the open shortest path first (OSPF) algorithm [5], excel in static environments. However, they face challenges in responding to frequent topology changes and dynamic traffic patterns, limiting their effectiveness in rapidly evolving network environments. Additionally, these algorithms struggle to optimize specific performance targets.

In this context, dynamic routing protocols including Ad hoc On-Demand Distance Vector (AODV) [6] and Destination-Sequenced Distance-Vector (DSDV) [7] have been developed to address these challenges within Mobile Ad Hoc Network (MANET). AODV adapts quickly to changing link conditions with minimal processing and memory overhead, establishing unicast routes only when necessary. By using destination sequence numbers, it ensures loop-free routing and overcomes the limitations of traditional distance-vector protocols, maintaining efficient network performance.

In contrast, DSDV enhances the Bellman-Ford algorithm [2] by periodically broadcasting routing information and incorporating sequence numbers to accommodate the dynamic environment of mobile ad hoc networks. This approach effectively mitigates loop issues and improves overall network reliability. Additionally, optimization algorithms are commonly employed to enhance the performance and efficiency of communication networks. Notably, the ant colony algorithm effectively addresses optical blocking issues and achieves optimized path configuration by integrating both local and global search methods [8,9]. By utilizing its simple and rapid local optimization steps, the greedy algorithm effectively solves communication network optimization problems and delivers robust solutions [10]. Additionally, Zhang applied game theory to analyze the interactions among various entities in communication networks, resulting in the development of a fair and efficient resource allocation mechanism [11]. These advancements highlight the diverse strategies employed to enhance network performance. Despite their effectiveness, traditional dynamic routing protocols encounter limitations in adaptability and efficiency as the complexity and diversity of application demands in modern internet environments continue to grow. Consequently, researchers are increasingly exploring advanced routing optimization techniques to overcome these challenges.

**Figure 1:**
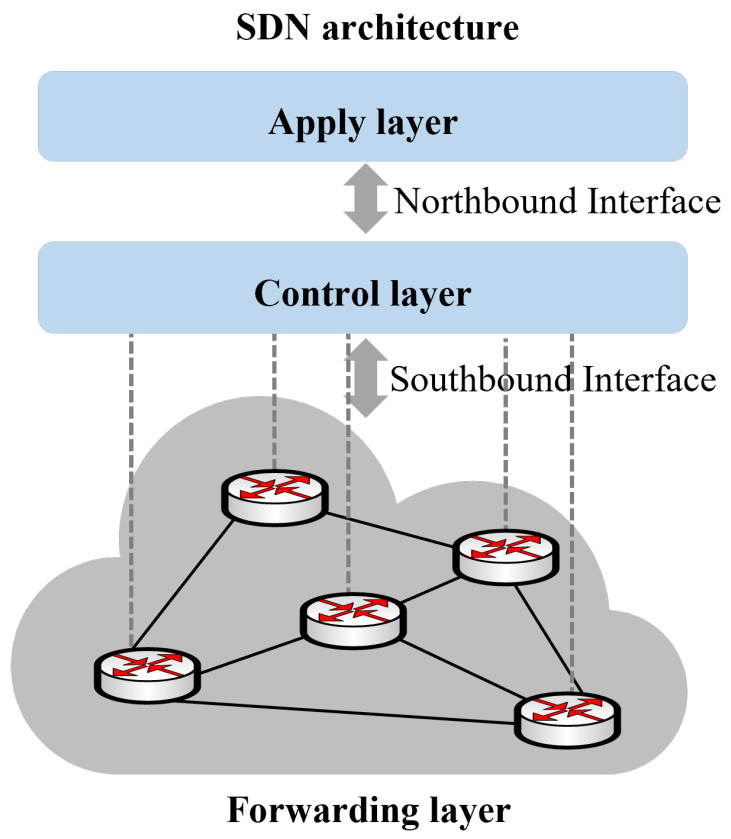
Software Define Network Architecture.

The advent of Software-Defined Networking (SDN) [12] has marked a significant evolution in routing optimization methods, shifting towards centralized management, dynamic adjustment, and network virtualization. SDN facilitates flexible and intelligent routing optimization through a centralized controller that monitors network status, computes optimal paths, and deploys routing policies, as shown in Figure 1. This separation of data and control has created new avenues for research in routing optimization. Within this framework, optimization techniques have been employed to enhance network performance and efficiency. These techniques include traffic engineering [13], machine learning [14], and multi-objective optimization [15]. Dynamic adjustment strategies are also used to adapt to changing network conditions. Sun et al. demonstrated the effectiveness of genetic algorithms in routing optimization. Nonetheless, genetic algorithms lack dynamic management mechanisms. They are complex to implement and tune, requiring extensive experimentation and optimization. Additionally, their real-time performance is inadequate for network environments that require rapid responses [16].

In recent years, Reinforcement Learning (RL) [17] has played a significant role in addressing complex problems and enhancing stability and adaptability. Awad et al. demonstrated that applying RL algorithms to compute optimal routing and deploying them on forwarding devices can enhance the performance and resource utilization efficiency of SDN networks [18]. Furthermore, Ye et al. proposed a novel destination-based traffic engineering solution named FlexEntry. This solution significantly reduces routing update overhead and optimizes network performance by combining RL with linear programming [19].

Deep Reinforcement Learning (DRL) [20], as an advanced form of RL, further enhances the modelling capability and decision-making accuracy in complex network environments. By employing deep neural networks to learn high-level abstract features, DRL can automatically extract and utilize regularities within data, thereby optimizing the routing decision process. This advancement enhances the intelligence and adaptability of SDN networks, addressing challenges related to dynamic changes and large-scale deployments, leading to more efficient resource allocation and improved network performance. Yu et al. implemented DQN in the SDN plane, but the Q-table consumes substantial memory, potentially affecting network performance [21]. Huang et al. introduced a routing optimization algorithm based on Dueling DQN by improving the Deep Q-Network (DQN) [20,22]. This algorithm integrates global network information from SDN with Dueling DQN to derive optimal forwarding routes. As a result, significant improvements in network throughput, latency reduction, and packet loss mitigation are achieved. Additionally, overall network performance and convergence speed are enhanced. Building on these advancements, the TIDE algorithm [23] incorporates Recurrent Neural Networks (RNN) [24] into DRL to refine routing strategies by leveraging temporal features. However, its convergence and exploration efficiency still require further enhancement to better meet the demands of highly dynamic networks.

Network topologies are inherently graph-based, and graph neural network (GNN) demonstrates superior generalization capabilities in handling graph-structured data. GNN can effectively model the complex relationships between nodes and edges, making it more suitable for routing optimization tasks compared to CNN and RNN [25,26]. The ability of GNN to aggregate and propagate information across the graph allows it to capture intricate dependencies that are critical for optimal path selection. This capability is essential for routing optimization, as GNN can dynamically adjust to changes in network topology, traffic patterns, and resource availability, making it more adaptable and efficient in optimizing routing decisions compared to traditional deep learning models. Information is iteratively aggregated and propagated across nodes. This allows each node’s representation to be updated based on its neighbors. As a result, a more accurate representation of the global network state is achieved. Rusek [27] introduced RouteNet, a novel network model based on GNN, which effectively predicts key performance indicators and supports efficient routing optimization. Similarly, Chen [28] developed a routing optimization solution that integrates DRL with GNN to address the limitations of existing methods in processing network topology information. Swaminathan [29] designed GraphNET, a GNN-based routing algorithm that utilizes global information from the SDN controller to predict paths with minimal average delay between source and destination nodes. This algorithm demonstrates impressive cross-topology generalization and robustness. Almasan [30] further advanced routing optimization by combining DRL and GNN to enhance data transmission efficiency in dynamically changing network environments. Although Jay [31] proposed a congestion control method that performs excellently in specific network scenarios, it still faces limitations in real-time optimization under dynamic topologies. To address these challenges, Almasan [32] introduced the Enero solution. This approach employs DRL to develop long-term routing strategies and enhances adaptability to dynamic network environments through the integration of GNN. Furthermore, local search algorithms are utilized to refine DRL results, ensuring both efficiency and low computational overhead. Their research demonstrates that the combination of DRL and GNN can effectively exploit network topology characteristics, thereby improving network resource utilization.

Despite the efficiency of using DRL to address routing problems, current approaches still face several challenges: (1) The focus is frequently placed on optimizing network traffic without simultaneously addressing multiple objectives. Researchers typically focus on optimizing link utilization without adequately considering and coordinating various communication resources, resulting in inefficient resource utilization. (2) Different users and applications have diverse communication requirements, and the variability in resource demands can result in imbalanced resource allocation during optimization. Routing optimization needs to be flexible to accommodate diverse user needs and quality of service requirements. Consequently, overall network performance may not be adequately improved, and specific user needs may not be effectively addressed.

To address the constraints above, we propose an Adaptive Routing algorithm with joint optimization of Multi-dimensional network Resources (AR-MR) based on the graph neural network. This algorithm efficiently adapts to dynamic communication network environments by integrating GNN and DRL. The main contributions of this paper are summarized as follows:

- This paper is the first to model multi-dimensional network resources, including bandwidth, delay, power consumption, and communication distance, as a Resource Tensor (RT). This approach addresses the limitations of traditional methods, which often fail to consider and efficiently utilize multi-dimensional resources in routing optimization comprehensively.
- To address the limitations of traditional routing optimization schemes in dynamic and complex network environments, the RT is introduced into the DRL framework. By integrating the RT, our method adapts more effectively to changing network conditions, providing a global perspective on resource allocation and significantly enhancing the algorithm’s adaptability and overall performance. This approach offers strong support for efficient resource management and optimization in complex network environments.
- A GNN-based adaptive routing joint optimization method for multi-dimensional network resources is proposed. This method utilizes GNN to adaptively learn resource weights, facilitating balanced optimization of multi-dimensional resources as network conditions change. It overcomes the limitations of existing models in adapting to dynamic resource changes. This enables the adaptive optimization of communication networks. As a result, overall network performance and resource utilization efficiency are significantly enhanced.

This paper is organized as follows. Section 2 describes the concept of routing optimization. Section 3 presents the proposed AR-MR method. Finally, Section 4 provides an analysis and discussion of the simulation results.

## 2. Preliminaries

In this section, the background knowledge and the issue need to be solved are briefly described.

### 2.1. Problem Statement

**Figure 2:**
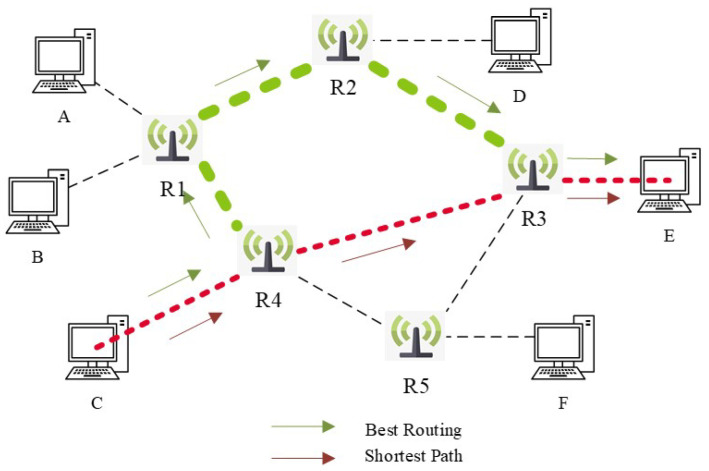
Communication Network. C to E: Bandwidth Requirements Data Streaming. In the communication network, although the red path has fewer hops, the green path is preferred for high-bandwidth data transmission due to its greater available bandwidth.

To state the problem clearly, we propose a network with six terminals and five routers, as shown in Figure 2. Suppose a data streaming needs to be transmitted from computer C to computer E. Traditional routing methods typically choose the shortest path [33] (as indicated by the red arrows in Figure 2 for packet transmission. This strategy implicitly assumes that physical distance is the dominant optimization objective. However, in practice, data packets often have specific requirements for bandwidth and delay, which means the shortest path is not always the optimal solution. When these multiple resource requirements are jointly considered, it was found that alternative paths, shown by the green arrows in Figure 2, provide better performance in terms of both bandwidth and delay compared to the shortest route. Although these alternative paths involve longer physical distances, they were selected for packet transmission due to their better alignment with bandwidth and delay requirements. This indicates that routing decisions are constrained by multiple network resources rather than a single metric.

This case illustrates that path selection in communication networks is determined not only by physical distance but also by factors including bandwidth and delay. Therefore, effective routing optimization must integrate these network resources to meet data transmission requirements. Accordingly, in this manuscript, we propose a multi-dimensional optimization approach to enhance network performance, ensuring efficient packet transmission while meeting specific quality of requirements.

### 2.2. Multi-Objective Joint Optimization

To efficiently address the issues above, it is imperative to consider the multi-dimensional resource requirements and constraints within the network environment. This encompasses not only bandwidth and delay but also factors such as distance and power consumption. Consequently, we introduce a multi-objective joint optimization problem, which involves balancing multiple objectives to select the optimal path. In the following sections, this multi-objective joint optimization problem is mathematically formulated. The relationships between various resources and performance indicators are systematically described. The optimization methods employed are also detailed.

Specifically, the multi-objective joint optimization problem can be defined as follows. In a network with *N* nodes and *E* edges, the goal is to find a path *S* from the source node *s* to the destination node *d* that minimizes the objective function below.

(1) $$\begin{array}{cc} \min_{S}(\sum_{(i,j)\in S}b\left(i,j\right) & +\sum_{(i,j)\in S}d\left(i,j\right)+\sum_{(i,j)\in S}p\left(i,j\right)+\sum_{(i,j)\in S}c\left(i,j\right))\\ s.t.\; & 0\leq B\left(e\right)\leq B_{\max}\left(e\right),\;\forall e\in S,\\ & 0\leq D\left(S\right)\leq D_{\max},\;\;\;\forall S,\\ & 0\leq P\left(e\right)\leq P_{\max}\left(e\right),\;\forall e\in S,\\ & 0\leq C\left(e\right)\leq C_{\max}\left(e\right),\;\forall e\in S. \end{array}$$

where *i* and *j* represent different nodes; Equation (1) aims to minimize the aggregated values of bandwidth utilization, delay, power consumption, and communication distance in the network, thereby enhancing the overall performance and resource utilization efficiency of the network. The constraints of the above optimization problem include: the Path *S* must be valid, meaning it should connect the source node to the destination node. Each edge in the path must meet several resource constraints. First, the bandwidth on each edge cannot exceed the maximum available bandwidth for that edge. Second, the total latency across the entire path must be less than or equal to the maximum allowed latency. Third, the power consumption for each edge must not surpass the maximum allowable power consumption. Additionally, the total communication distance across all links on the path must not exceed the predetermined maximum distance constraint for the network.

By adjusting the resource weight coefficients learned via GNN, varying communication requirements can be accommodated. The four optimization objectives (e.g., distance, latency, bandwidth, and power consumption) are balanced through this adjustment. As a result, optimal path selection is achieved. These four dimensions are chosen because they capture the most critical link-level resources affecting routing performance. Other factors like packet loss, congestion, and node load are indirectly reflected through bandwidth, delay, and power. Limiting the Resource Tensor to these core dimensions keeps the model compact and stable, while remaining extensible for additional metrics in future applications.

## 3. Adaptive Routing Algorithm of Multi-Dimensional Network Resources

Current DRL-based approaches predominantly focus on optimizing link utilization [34]. However, this single-indicator optimization strategy proves inadequate in a complex and dynamic network environment with time-varying traffic demands and device status fluctuations, as it neglects other crucial performance indicators including end-to-end delay, packet loss rate, and traffic fairness [32]. Such one-sided optimization may even lead to unbalanced network operation, where some links are overloaded due to excessive focus on utilization while others remain underutilized, further deteriorating overall network performance. While DRL-based methods can provide optimization benefits in specific contexts, their flexibility and adaptability in practical applications remain limited because most of them are trained based on fixed network scenarios and lack effective generalization capabilities when facing sudden changes in network topology or traffic intensity. To address these limitations, we propose a model with enhanced generalization and adaptability, aiming to achieve more comprehensive optimization across dynamic and complex network scenarios by integrating multi-dimensional network resource indicators into the decision-making process and constructing a more accurate network state representation mechanism.

This section provides a detailed overview of the adaptive routing solution for the joint optimization of multi-dimensional network resources, which is designed to address the aforementioned limitations of existing DRL-based routing methods. Initially, we explain the method for constructing the RT since the accurate representation of network resources is the foundation of adaptive routing decision-making and multi-objective optimization. Subsequently, we introduce the adaptive routing solution for the joint optimization of multi-dimensional network resources, elaborating on how the proposed solution utilizes the constructed RT to realize dynamic adjustment of routing strategies and comprehensive optimization of network performance.

### 3.1. Resource Tensor Construction

In the field of routing optimization, graph models are commonly used to represent nodes and their connections in a communication network. A communication network can be defined as G=(V,E), where *V* is the set of nodes, and *E* is the set of edges. Nodes typically represent network devices, including computers, routers, switches, etc., while edges represent the connections between these network devices. Let the set of nodes be $V=\{v_{1},v_{2},\ldots ,v_{n}\}$, where *n* is the number of nodes. Each node $v_{i}$ is represented by a feature vector $X_{i}$, which contains the attribute information of node $v_{i}$. Let the set of edges be $E=\{\left(v_{i},v_{j}\right),\left(v_{k},v_{l}\right),\ldots \}$, where $\left(v_{i},v_{j}\right)$ indicates a connection between nodes $v_{i}$ and $v_{j}$. Each edge $\left(v_{i},v_{j}\right)$ is represented by a feature vector $W_{ij}$. By graph modelling, the network topology, device interconnections, and performance indicators are abstracted into a graphical structure, facilitating more effective optimization of the communication network. However, traditional graph models capture only topology and single-dimensional edge features, failing to reflect the full state of network resources and limiting support for multi-objective routing optimization in dynamic scenarios.

**Figure 3:**
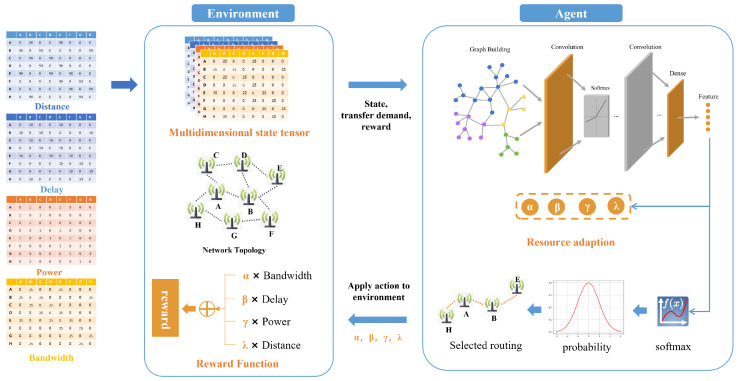
Figure of Multi-domain Network Representation Learning Algorithm Architecture.

In this work, four dimensions of communication resources including bandwidth, end-to-end delay, power consumption, and communication distance, are incorporated to construct the RT. These four dimensions are selected as core indicators of routing performance: bandwidth determines transmission capacity, end-to-end delay affects real-time quality, power consumption is key for energy efficiency, and communication distance relates to signal stability and transmission cost. Specifically, these four indicators are employed to model the network state, as shown in Figure 3. This multi-dimensional approach provides a more accurate representation of the network performance. Unlike the single-indicator modeling of existing DRL-based approaches, the multi-dimensional RT captures comprehensive network resource characteristics and their real-time dynamics. It offers more detailed information to optimization algorithms, thereby improving network resource management and scheduling and laying a solid foundation for the adaptive routing decision-making of the proposed AR-MR solution.

Assuming there are *n* nodes in the network, the resource tensor T can be represented as a four-dimensional tensor $T_{ijk}$, where *i* and *j* denote the node indices, and *k* represents the resource dimension. The resource tensor is defined as

(2) $$T_{ijk}=\left\{\begin{array}{cc} B_{ij}, & \mathrm{if}\;k=1\\ D_{ij}, & \mathrm{if}\;k=2\\ P_{ij}, & \mathrm{if}\;k=3\\ C_{ij}, & \mathrm{if}\;k=4 \end{array}\right.$$

where $B_{ij}$ is the bandwidth value between node *i* and node *j*, measured in Mbps; $D_{ij}$ is the end-to-end delay between node *i* and node *j*, measured in milliseconds; $P_{ij}$ is the power consumption between node *i* and node *j*, measured in watts; and $C_{ij}$ is the physical distance between node *i* and node *j*, measured in meters. Each tensor component is updated in real time based on actual network state, ensuring the RT accurately reflects resource dynamics and provides the DRL-based routing module with timely, up-to-date network information.

We use the RT as the environment state in DRL, which not only provides a comprehensive and accurate representation of the network state but also facilitates multi-objective optimization. As a result, it enhances the dynamic adaptability of decision-making and the robustness of the system. This effectively overcomes the limited adaptability of existing DRL-based routing methods in dynamic, complex networks and enables joint optimization of multi-dimensional performance indicators rather than single link utilization.

### 3.2. Resource Adaptive Module Based on GNN

In DRL, the reward function plays a critical role by defining explicit learning objectives that guide the agent’s exploration of the environment and the refinement of its decision-making process. Traditionally, the reward function has been employed to quantify changes in maximum link utilization. This method focuses on minimizing network utilization to enhance network performance and improve resource efficiency.

In our approach, the reward function for the DRL agent includes four components: bandwidth, delay, power consumption, and communication distance. Given that the significance of each resource can vary under different network conditions, we introduce a weight-adaptive mechanism. This method allows the agent to dynamically adjust the weight of each component in the reward function based on real-time environmental changes and specific requirements. Such adaptability enables the agent to more effectively address diverse communication needs and network conditions, thereby facilitating the joint optimization of multi-dimensional network resources. Furthermore, in scenarios with sparse rewards, this weight-adaptive approach enhances the agent’s ability to leverage limited reward signals more efficiently for learning.

Initially, the RT is passed to adjacent neighboring nodes for aggregation and updating, facilitating the sharing and efficient transmission of communication network information.

(3) $$m_{j\to i}^{\left(k\right)}=\sigma \left(W_{m}\left[h_{j}^{\left(k-1\right)},h_{i}^{\left(k-1\right)},e_{ij}\right]+b_{m}\right)$$

(4) $$m_{i}^{\left(k\right)}=\sum_{j\in N(v_{i})}m_{j\to i}^{\left(k\right)}$$

(5) $$h_{i}^{\left(k\right)}=\mathrm{GRU}\left(h_{i}^{\left(k-1\right)},m_{i}^{\left(k\right)}\right)$$

where *i* and *j* denote the target node and neighbor node identifiers, respectively, while *k* represents the layer of message passing. $m_{j\to i}^{\left(k\right)}$ denotes the message from neighbor node *j* to target node *i* at layer *k*. $h_{i}^{\left(k-1\right)}$ and $h_{j}^{\left(k-1\right)}$ represent the representations of the target node *i* and neighbor node *j* at layer k−1, respectively. $e_{ij}$ denotes the edge features. GRU is adopted to update node representations because it effectively captures temporal dependencies and stabilizes information propagation during iterative message passing while maintaining lower computational overhead compared to LSTM. σ is the SELU activation function, SELU is selected due to its self-normalizing property, which improves training stability and convergence in deep architectures, and $W_{m}$ and $b_{m}$ are the learned weight matrix and bias, respectively. $m_{i}^{\left(k\right)}$ represents the aggregated representation of target node *i* after collecting messages from neighbor nodes *j* at layer *k*. $N(v_{i})$ denotes the set of neighbor nodes of $v_{i}$. $h_{i}^{\left(k\right)}$ represents the updated representation of the target node *i* at layer *k*, with GRU [35] used for updating node representations. The key hyperparameters, including hidden state dimensions, number of message-passing layers, and activation choices, were determined through empirical validation to balance performance and computational efficiency.

Subsequently, dimensionality reduction is performed on the aggregated high-dimensional features to obtain adaptive weights for each resource dimension as follows

(6) $${\hat{h}}_{i}^{\left(k\right)}=\sigma^{\prime }\left(W^{\prime }\cdot h_{i}^{\left(k\right)}+b^{\prime }\right)$$

where ${\hat{h}}_{i}^{\left(k\right)}$ represents the node features mapped to the lower-dimensional space with a dimension of *d*; $h_{i}^{\left(k\right)}$ denotes the updated representation of node *i* at layer *k*, with a dimension of 5; $W^{\prime }$ represents the learned weight matrix of dimension 5×d, and $b^{\prime }$ represents the learned bias of dimension 5; $\sigma^{\prime }$ is the SELU activation function.

By adaptively learning resource weights through neural networks, key features are extracted while computational complexity is reduced. This approach also enhances the model’s adaptability to varied network conditions. The introduction of adaptive weights allows the DRL model to flexibly adjust the weights of different resource dimensions in complex and changing network environments, leading to more accurate multi-objective optimization. Furthermore, the adaptive weight mechanism directs the agent towards optimal strategies more efficiently in scenarios with sparse rewards, thereby significantly boosting the performance of DRL in routing optimization.

Subsequently, the reward function is constructed based on the adaptive weights of each resource dimension to achieve multi-objective joint optimization. The specific formula is as

(7) R=α·B(i,j)+β·D(i,j)+γ·P(i,j)+δ·C(i,j)

where *R* represents the reward function, *i* and *j* denote nodes in the network, B(i,j) represents bandwidth, D(i,j) represents delay, P(i,j) represents power consumption, and C(i,j) represents communication distance. α, β, γ, and δ are the weight coefficients for each resource. This construction method enables the reward function to dynamically reflect the significance of each resource dimension according to varying network conditions. By incorporating diverse resource constraints and performance indicators during the optimization process, this approach enhances the agent’s decision-making abilities in complex network environments. Additionally, greater flexibility and efficiency are introduced into the optimization process, better aligning with the needs of different communication scenarios in practical applications. The adaptive weights further enhance the robustness and adaptability of the reward function across various contexts, improving the overall effectiveness of network optimization.

### 3.3. Adaptive Routing for Joint Optimization

This section introduces the Adaptive Routing for Joint Optimization of Multi-Dimensional Network Resources, which is based on DRL and integrates both the multi-dimensional RT and the GNN-based resource adaptive module, as shown in Figure 3. To address the limitations of existing DRL-based routing methods in dynamic network scenarios and respond to the reviewer’s requirement on clarifying the workflow logic the workflow of this method mainly includes three core steps: constructing the state representation, defining the action space, and formulating the reward function. Each step is closely linked to the previous modules to ensure the overall logical coherence of the proposed solution.

Firstly, the environment state is constructed, incorporating the RT and network topology. As the foundation of DRL agent decision-making the state construction fully leverages the multi-dimensional resource information contained in the RT to accurately reflect the real-time network status which addresses the reviewer’s concern about the rationality of state representation. When the agent performs an action by executing a new routing strategy based on the current state, the RT is updated, leading to a change in the environment state. This real-time update mechanism ensures that the agent can perceive the dynamic changes of the network in a timely manner laying a foundation for adaptive routing decision-making.

Subsequently, the RT is input into the GNN for feature extraction and dimensionality reduction, resulting in low-dimensional resource features. These features are then combined with those from the previous time step, updated across the nodes, and utilized to determine the probability distribution for the agent’s actions. Based on this distribution, the agent devises the optimized routing strategy.

Simultaneously, the GNN evaluates the significance of resources and adaptively assigns weights to each resource in the reward function. As the agent executes an action, the environment state is updated accordingly, and feedback is provided to the agent via reward signals. This feedback mechanism guides the agent in refining its strategy and optimizing future decisions.

During interaction with the environment, the Actor-network generates training samples, which include link state information, current actions, rewards, and resource requirements. These samples are stored in the experience replay buffer. In each training iteration, samples are drawn from the experience replay buffer and used to generate learning samples for the neural network, creating several sets of sample information. The neural networks continuously update their parameters through learning and training on these samples to achieve ongoing optimization. This process, which involves continuous interaction and policy adjustment, enables the agent to achieve joint optimization of multi-dimensional resources in complex and dynamic communication networks. As a result, network performance and resource utilization efficiency are significantly enhanced.

We thoughtfully designed the loss functions to further enhance the agent’s learning efficiency and stability. In DRL, loss functions are critical because they directly influence the convergence speed and overall performance of the model. The following sections will detail the design principles of each key loss function and their specific roles in the training process. Each loss function is designed for a specific training objective to ensure that the model can stably learn optimal routing strategies.

First, the policy loss is used to train the Actor network. The policy loss measures the difference between the current policy and the old policy and evaluates the effectiveness of the selected actions using the advantage function. We employ the policy gradient method and apply clipping to the policy loss to prevent overly aggressive updates, thereby maintaining stability during training. The clipping operation is a key design to avoid model divergence which addresses the reviewer’s concern about training stability. The specific formula for the policy loss is as

(8) $$L_{p}=E\left[\max(-r_{t}\cdot A_{t},-\mathrm{clip}\left(r_{t},1-\epsilon ,1+\epsilon \right)\cdot A_{t})\right]$$

where $A_{t}$ is the advantage function, which measures the superiority of the action $a_{t}$ relative to the average action. The policy ratio $r_{t}$ is defined as

(9) $$r_{t}=\frac{\pi_{\theta }\left(a_{t}|s_{t}\right)}{\pi_{\theta_{\mathrm{old}}}\left(a_{t}|s_{t}\right)}.$$

Then, entropy loss is introduced to encourage exploration in policy exploration. The entropy loss penalizes overly deterministic policies, ensuring that the Actor-network maintains diversity during training, thereby avoiding suboptimal solutions. This effectively solves the problem of insufficient exploration of existing DRL-based routing methods which are prone to falling into local optimal solutions and responds to the reviewer’s suggestion on improving the model’s optimization ability. The formula for calculating the entropy loss is as

(10) $$L_{e}=-\lambda \cdot \sum_{a}\pi_{\theta }\left(a|s\right)\log\pi_{\theta }\left(a|s\right)$$

where λ is a coefficient that controls the strength of entropy regularization and the value of λ is determined through empirical validation to balance the exploration and exploitation of the agent. $\pi_{\theta }\left(a|s\right)$ represents the probability of selecting action *a* in state *s*.

For the Critic network, we use value function loss, which evaluates the difference between the Critic network’s predicted state value and the actual return using MSE. The specific formula is as

(11) $$L_{c}=E\left[{\left(R_{t}-V\left(s_{t}\right)\right)}^{2}\right]$$

where $R_{t}$ represents the actual return, and $V(s_{t})$ is the state value predicted by the Critic network.

Finally, we combine the policy loss and entropy loss of the Actor-network to form the total Actor loss:

(12) $$L_{a}=L_{p}+L_{e}$$

By combining the loss functions of the Actor and Critic networks, we derive the total loss used to optimize the entire model. The formula for the total loss is as

(13) $$L_{total}=L_{a}+L_{c}$$

This total loss function integrates the objectives of both networks, ensuring that the model learns to make accurate decisions while maintaining stability during training. By carefully balancing the contributions of the Actor and Critic losses, the model can effectively navigate complex environments and achieve optimal performance in routing optimization scenarios. The derived total loss serves as a robust foundation for improving overall network efficiency and adaptability in dynamic conditions. This loss function design fully considers the training needs of both Actor and Critic networks and effectively addresses the reviewer’s concerns about model training stability and optimization effectiveness.

## 4. Experimental Setup and Analysis

In this section, we conduct experiments with different network topologies and compare the AR-MR method with other representative approaches to evaluate the performance of our proposed method.

### 4.1. Experimental Setup

The experimental hardware environment includes a computer equipped with an Intel Core i5-9400F CPU (Intel, Santa Clara, CA, USA) and an NVIDIA^®^ GeForce RTX 2060 Ti GPU (NVIDIA, Santa Clara, CA, USA), running the Ubuntu 20.04 LTS operating system. The software environment utilizes Python 3.8 as the programming language and TensorFlow 2.6.0 as the deep learning framework. We specifically employed the Actor-Critic framework in DRL to train the model with consistent settings as the proposed network structure.

**(1) Implementation:** In our architecture, both the Actor and Critic networks share identical structures, with the Actor incorporating an advanced GNN. The information passing layer includes a fully connected neural network that transforms the input network resource features into a link hidden state with a dimension of 20, utilizing SELU as the activation function. The readout layer consists of three fully connected layers and two SELU activation functions. During forward propagation, the link hidden state transitions from the update layer to the readout layer, undergoing both linear and nonlinear transformations through these layers. Each neuron applies specific weight combinations and the activation function to the state, gradually mapping the network state to the output value range. SELU is adopted for its self-normalizing property to stabilize training and accelerate convergence. This approach ensures an accurate representation of the network and the generation of optimized strategies through efficient feature extraction and mapping.

**(2) Datasets:** The network topologies used in training and testing are separate shown in Table 1 and Table 2. We selected three network topologies from the TopologyZoo dataset [36] for model training and utilized two additional topologies for evaluation. The test set includes both small and large scale topologies to verify generalization ability across different network scales. The initial RT is generated based on a uniform distribution to ensure data diversity and representativeness, allowing us to test the model’s performance and robustness across different network environments. This approach facilitates a thorough assessment of the model’s adaptability and optimization effectiveness in various actual network scenarios. This design supports comprehensive validation of the model’s adaptability in realistic network scenarios.

**(3) Comparison Algorithm:** We compared the AR-MR algorithm with the following three methods

AODV [6] dynamically establishes routes by broadcasting route request information as needed, followed by route reply messages sent by the destination node or nodes with known paths.

DSDV [7] maintains a routing table and updates it through a periodic exchange of updated information, allowing nodes to remain aware of changes in the network topology and update their routing tables accordingly.

Enero [25] integrates DRL with GNNs and employs local search algorithms to refine routing configurations.

**Figure 4:**
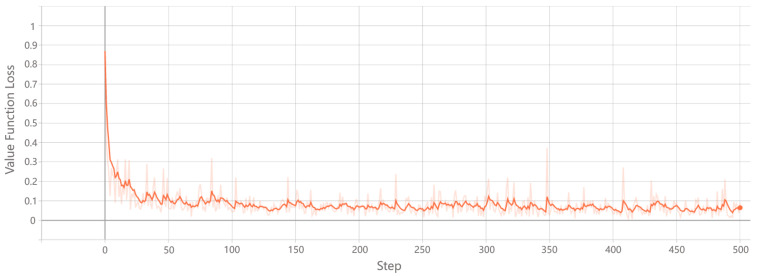
Value Function Loss. Convergence of value function loss over training iterations, demonstrating rapid decrease and eventual stabilization, indicating effective model learning.

**Figure 5:**
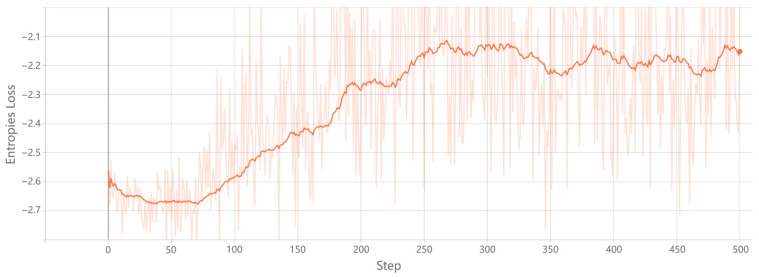
Entropy Loss. The absolute value of the entropy loss decreases over training, indicating reduced exploration and a gradual shift toward exploitation.

**(4) Training:** The training process iteratively updates the network’s parameters to minimize the value function loss, improving the model’s accuracy in predicting state values. As shown in Figure 4, the loss initially starts at a high level, near 1, and decreases rapidly as learning progresses. Eventually, the loss stabilizes, fluctuating between 0.05 and 0.1, indicating effective minimization of prediction errors. This trend demonstrates successful convergence, reflecting the model’s improved capacity to estimate state values accurately. In addition, Figure 5 illustrates the entropy loss during training. Initially, the entropy is low, indicating a focus on exploitation. As training proceeds, the entropy increases, promoting exploration and preventing premature convergence to suboptimal solutions. Over time, the entropy stabilizes, suggesting the model has achieved a balance between exploration and exploitation. This balanced behavior ensures stable and effective policy learning. These results confirm that the model effectively learns optimal routing policies while maintaining flexibility to explore alternative routes.

**(5) Simulation Platform:** In our research, we utilized the OMNeT++ (6.3.0) network simulation tool to evaluate the proposed algorithm. OMNeT++ (6.3.0) is a framework specifically designed for network modelling and simulation, offering a robust set of network modules and simulation capabilities that facilitate the modelling and simulation process in the field of network research. We selected OMNeT++ (6.3.0) for its mature module library, advanced network modelling features, and strong community support. In the simulation experiments, we configured parameters including communication duration, node count, and communication model to simulate the data transmission processes of different routing algorithms in a real network. We then compared their performance differences across different scenarios. These experiments allowed us to thoroughly evaluate the effectiveness and performance of our proposed algorithm under diverse network conditions.

In the experiments, parameters including simulation time, node count, and communication models were configured to simulate the data transmission processes of different routing algorithms in a real network. Their performance differences were then compared. Specifically, simulations were conducted under congested and complex network environments that exceed the handling capabilities of existing protocols. The simulated network experienced frequent topology changes, varying traffic loads, and demands for multidimensional resources including bandwidth, delay, power consumption, and communication distance. These settings reflect realistic challenges that traditional protocols cannot effectively handle.

### 4.2. Experimental Analysis

In this section, we compare the performance of the AR-MR algorithm with other baseline algorithms under different bandwidth conditions. The results indicate that the AR-MR algorithm offers significant advantages across all evaluation indicators. Specifically, the bit error rate and end-to-end delay are effectively reduced by AR-MR, which also outperforms AODV, DSDV, and Enero in terms of packet transmission quantity. These advantages stem from the integration of multi-dimensional resource tensor modeling and GNN-based adaptive weight mechanism. We will now present a detailed analysis of each indicator and discuss the underlying reasons for these differences to validate the effectiveness of the AR-MR algorithm further.

**Figure 6:**
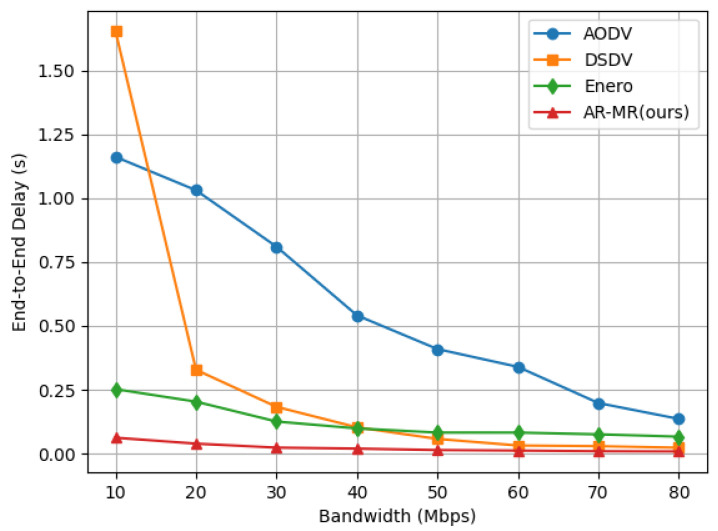
End-to-End Delay on the EBackbone Topology.

The end-to-end delay of the 4 methods decreases significantly with increasing bandwidth, as shown in Figure 6. This reduction occurs because faster data transmission speeds and decreased network congestion improve overall performance. DSDV exhibits the highest delay at low bandwidth. This is due to the overhead of routing table initialization and periodic updates. These processes consume a considerable amount of bandwidth and time under low bandwidth conditions. As bandwidth increases AODV’s delay exceeds DSDV because its broadcast-based route discovery generates more control overhead in high-bandwidth scenarios. Enero achieves lower end-to-end delay compared to AODV and DSDV by balancing the traffic load, which indirectly reduces delay. In contrast, AR-MR consistently maintains the lowest end-to-end delay across different bandwidths. Specifically, at 10 Mbps bandwidth, AR-MR reduces delay by 94.64%, 96.24%, and 75.34% compared to AODV, DSDV, and Enero, respectively. This is due to our intelligent routing algorithm’s optimization of resource allocation and congestion control, which enhances bandwidth utilization, mitigates network congestion, and dynamically adjusts traffic to prevent bottlenecks and retransmissions. As a result, transmission delay is reduced, and data transmission efficiency is improved. The dynamic adaptation of the network bandwidth changes ensures stable low-delay performance which is critical for delay-sensitive applications.

**Figure 7:**
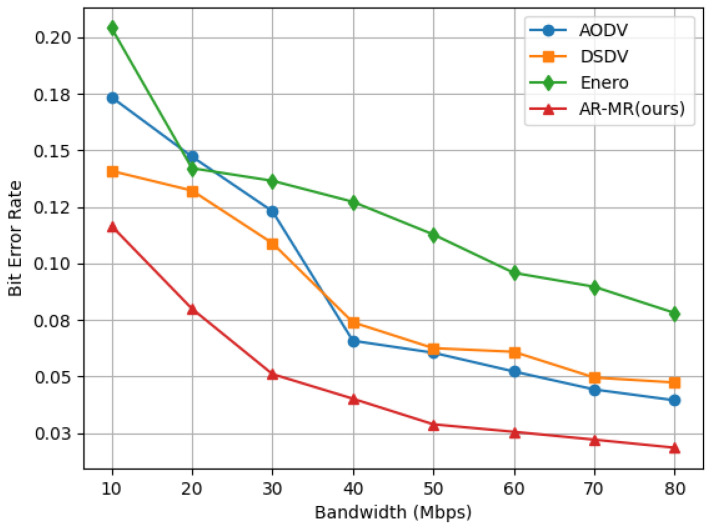
Bit Error Rate on the EBackbone Topology.

AR-MR exhibits satisfactory performance in the joint optimization process even when the bit error rates is not a primary optimization objective, as shown in Figure 7. At 10 Mbps bandwidth, AR-MR achieves reductions in bit error rate of 32.80%, 17.24%, and 42.87% compared to AODV, DSDV, and Enero, respectively. At a bandwidth of 40 Mbps, the dynamic changes in the network significantly impact performance. The fixed update mechanism in DSDV results in increased control overhead and conflicts. This leads to poorer performance when compared to AODV. At higher bandwidths, AODV’s performance deteriorates due to network conflicts generated by its route discovery process. The performance degradation of AODV at 40 Mbps is caused by its broadcast-based route discovery and frequent route maintenance which increase control overhead and contention under high traffic loads. Enero primarily focuses on optimizing link utilization and lacks a comprehensive view of other critical factors, making it less effective in reducing bit error rates. In contrast, AR-MR optimizes both path selection and resource allocation, effectively mitigating congestion and interference, which reduces packet loss and errors during transmission. Lower bit error rate not only enhance user experience but also decrease the additional burden caused by packet retransmissions, thus improving overall network efficiency. The improved stability and lower error rates of AR-MR translate into reduced retransmissions, improved QoS, and enhanced reliability for delay-sensitive and bandwidth-intensive applications. The deterioration of AODV performance at 40 Mbps arises from its broadcast-based route discovery and frequent route maintenance, which increase control overhead and contention under high traffic loads. In contrast, AR-MR dynamically balances traffic and avoids excessive control signaling, resulting in more stable performance. This reduction in bit error rate further reinforces the reliability of AR-MR, ensuring higher quality service in practical applications. Future research will focus on investigating bit error rate performance under different environments and conditions. The algorithm will be further refined to maintain a low bit error rate and high efficiency in more complex network scenarios.

**Figure 8:**
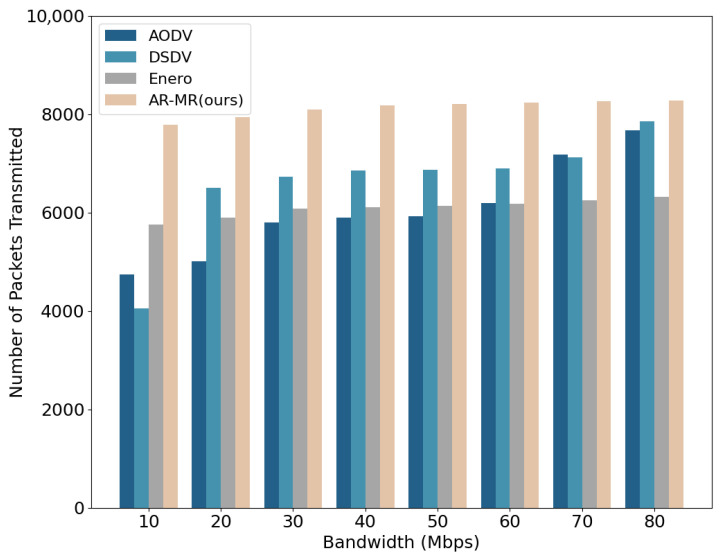
Number of Packets Transmitted on the EBackbone Topology.

A comparison of the number of packets transmitted under varying bandwidth conditions among AODV, DSDV, Enero, and AR-MR is shown in Figure 8. At 10 Mbps bandwidth, AR-MR increases the number of transmitted packets by 64.44%, 92.34%, and 35.47% compared to AODV, DSDV, and Enero, respectively. Clearly, AR-MR demonstrates significant advantages in data transmission efficiency. This is primarily due to the optimized allocation of communication resources, which leads to more effective utilization of network resources and reduced packet loss and retransmissions. Moreover, AR-MR can adaptively adjust routing strategies to cope with dynamic network environments, thereby maintaining high transmission efficiency. In contrast, DSDV generally ranks second in packet transmission efficiency under most bandwidth conditions, but it performs worse than AODV at 10 M bandwidth. This is due to DSDV’s reliance on periodic routing table updates, which consume substantial bandwidth and negatively impact data packet transmission efficiency. Enero, despite optimizing link utilization, fails to account for resource competition among different traffic flows. This oversight can lead to delays in packet transmission in high-competition environments, resulting in a lower overall packet transmission count. These differences highlight that multi-dimensional resource joint optimization of AR-MR is more effective in utilizing network resources and enhancing communication efficiency than single-indicator optimization methods.

**Figure 9:**
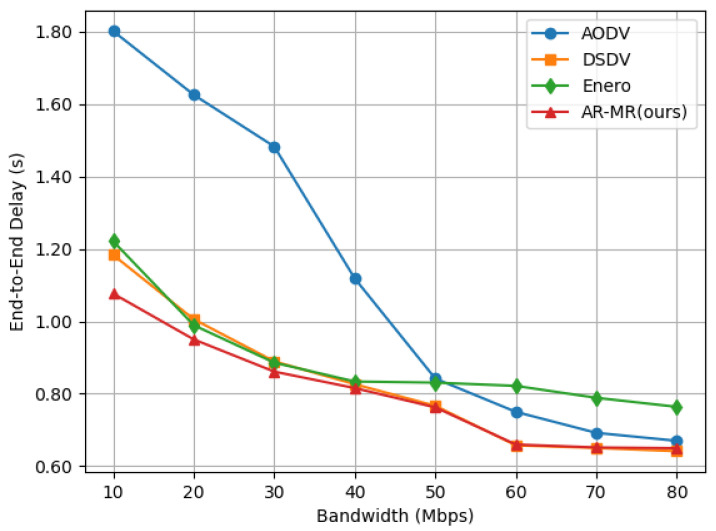
End-to-End Delay on the MegaNet Topology.

**Figure 10:**
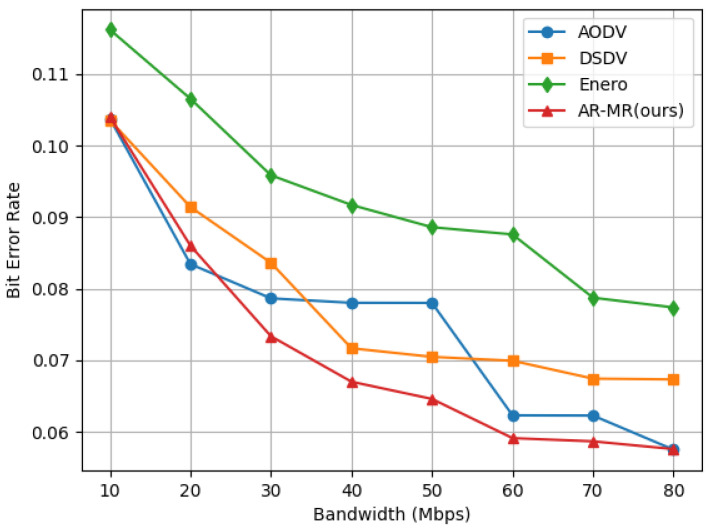
Bit Error Rate on the MegaNet Topology.

**Figure 11:**
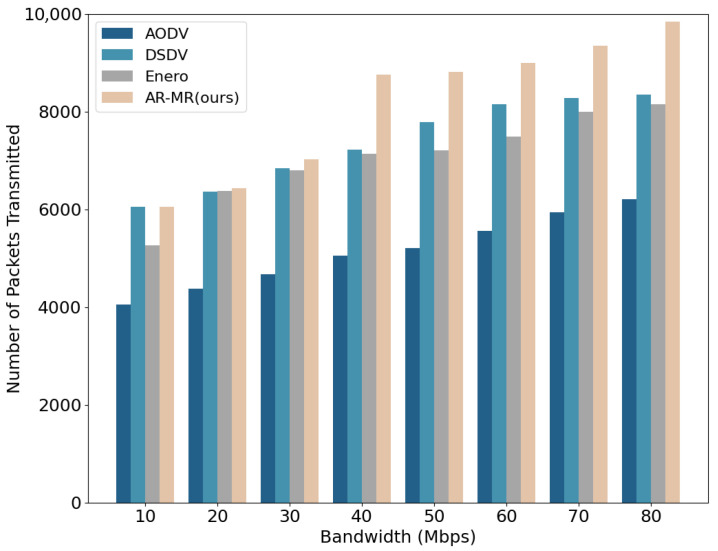
Number of Packets Transmitted on the MegaNet Topology.

In larger-scale network topologies, our simulations and evaluations, as shown in Figure 9, Figure 10 and Figure 11, demonstrate that AR-MR outperforms the compared methods in terms of delay, bit error rate, and packet transmission. This enhanced performance is primarily attributed to our optimized resource utilization, dynamic adaptation capabilities, and low control overhead. Specifically, AR-MR effectively coordinates multi-dimensional network resources, ensuring high transmission efficiency across diverse network conditions. Moreover, AR-MR adaptively adjusts routing strategies to respond to dynamic network environments. Optimal paths are promptly selected, and resources are efficiently allocated, even amidst frequent topology changes. This ensures stable network performance. Furthermore, unlike DSDV, which requires periodic routing table updates, AR-MR reduces control overhead, thereby freeing up more bandwidth for actual packet transmission. This reduction in control overhead is especially crucial in large-scale networks, where excessive overhead can substantially impair overall performance. The linear computational complexity of AR-MR with respect to the number of edges during inference ensures it maintains scalability in large-scale networks.

**Figure 12:**
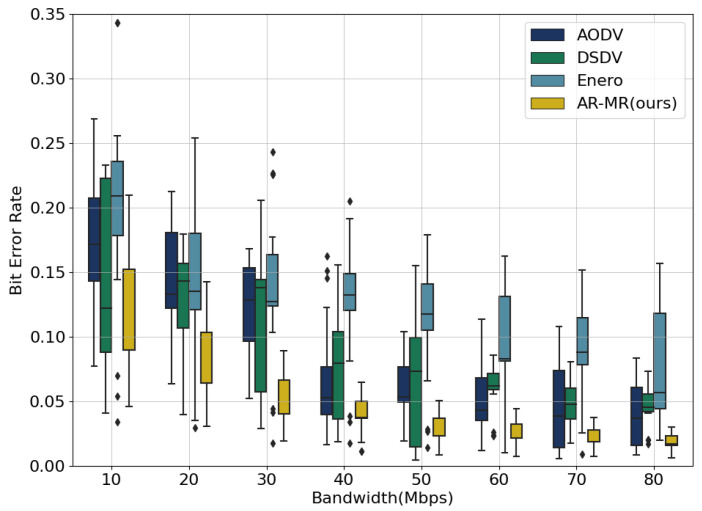
Bit Error Rate for EBackbone.

**Figure 13:**
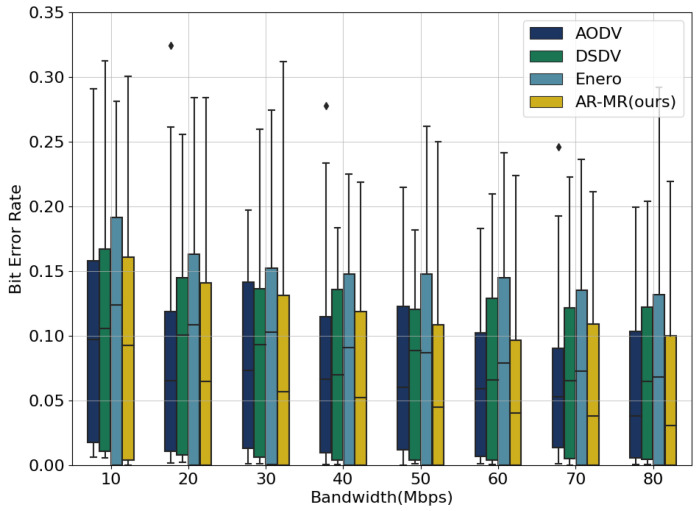
Bit Error Rate for MegaNet.

A comparison of the bit error rate box plots for small and large networks reveals consistently lower bit error rates and greater stability achieved by AR-MR across both network scales. This is especially evident in small networks, as shown in Figure 12. As bandwidth increases, the bit error rate generally decreases for all methods. However, in large-scale networks, shown in Figure 13, the abundance of network resources, including bandwidth and routing paths, leads to more balanced resource allocation and utilization. Consequently, the differences in bit error rate due to resource competition diminish, resulting in reduced performance disparities among the four methods. Although AR-MR maintains its advantage in large-scale networks, the performance gap compared to small-scale networks is less pronounced. This reduced performance gap is due to increased path diversity and abundant resources in large networks, which alleviate network congestion and lessen the benefits of multi-dimensional optimization. The computational complexity of AR-MR grows approximately linearly with the number of edges during inference, and offline training with centralized controllers can effectively amortize this cost in large SDN deployments. This suggests that while AR-MR adapts well to different network sizes, there remains potential for further optimization in larger-scale networks.

In summary, our work demonstrates that the AR-MR algorithm outperforms AODV, DSDV, and Enero in terms of bit error rate, end-to-end delay, and packet transmission count across various network scales and bandwidth conditions. Specifically, the AR-MR algorithm achieves a lower bit error rate and end-to-end delays while exhibiting outstanding stability. While AR-MR’s performance advantage is more pronounced in small-scale networks, it continues to hold a competitive edge in large-scale networks, albeit with slightly reduced effectiveness. These results validate the robustness and adaptability of AR-MR across different network conditions confirming its potential for efficient and reliable network communication in practical scenarios.

## 5. Conclusions

This paper presents the AR-MR approach for multi-dimensional resource-aware routing optimization, integrating GNN and DRL frameworks to effectively address dynamic network environments. Through comprehensive simulations on the OMNeT++ platform, AR-MR demonstrated superior performance compared to AODV, DSDV, and Enero, significantly reducing end-to-end delay and bit error rate while improving packet transmission efficiency. The results confirm that AR-MR is particularly effective in large-scale network topologies, where it exhibits clear advantages in managing complex network states and mitigating resource competition impacts. This approach not only achieves the research objective of optimizing routing decisions by considering multiple resource dimensions but also enhances the adaptability and robustness of communication networks in practical deployments. The findings underscore AR-MR’s potential to significantly improve network performance in real-world applications, providing a strong foundation for future optimization efforts in more diverse and large-scale network scenarios with dynamic characteristics. Future work will focus on refining the model’s adaptability to further complex network conditions and disruptions, and exploring real-time implementations in live communication networks.

## Figures and Tables

**Table 1:** Network Topologies Used in Training.

Topology	Nodes	Edges
Goodnet	17	62
BtAsiaPac	20	62
Garr199905	23	50

**Table 2:** Network Topologies Used in Testing.

Topology	Nodes	Edges
EBackbone	20	60
MegaNet	54	72

## Data Availability

The original contributions presented in this study are included in the article. Further inquiries can be directed to the corresponding author.
